# MXene-Based Chemo-Sensors and Other Sensing Devices

**DOI:** 10.3390/nano14050447

**Published:** 2024-02-28

**Authors:** Ilya Navitski, Agne Ramanaviciute, Simonas Ramanavicius, Maksym Pogorielov, Arunas Ramanavicius

**Affiliations:** 1 Department of Nanotechnology, State Research Institute Center for Physical Sciences and Technology (FTMC), Sauletekio av. 3, LT-10257 Vilnius, Lithuania; ilya.nov42@gmail.com; 2 Department of Physical Chemistry, Faculty of Chemistry and Geosciences, Institute of Chemistry, Vilnius University, Naugarduko 24, LT-03225 Vilnius, Lithuania; agne.ramanaviciute@gmail.com; 3 Department of Organic Chemistry, State Research Institute Center for Physical Sciences and Technology, Saulėtekio av. 3, LT-10257 Vilnius, Lithuania; simonas.ramanavicius@ftmc.lt; 4 Biomedical Research Centre, Sumy State University, 2, Kharkivska Str., 40007 Sumy, Ukraine; 5 Institute of Atomic Physics and Spectroscopy, University of Latvia, 3 Jelgavas St., LV-1004 Riga, Latvia

**Keywords:** MXenes, strain sensors, pressure sensors, temperature sensors, humidity sensors, gas sensors, 2D nanomaterials, volatile compounds, MAX phase, energy storage

## Abstract

MXenes have received worldwide attention across various scientific and technological fields since the first report of the synthesis of Ti_3_C_2_ nanostructures in 2011. The unique characteristics of MXenes, such as superior mechanical strength and flexibility, liquid-phase processability, tunable surface functionality, high electrical conductivity, and the ability to customize their properties, have led to the widespread development and exploration of their applications in energy storage, electronics, biomedicine, catalysis, and environmental technologies. The significant growth in publications related to MXenes over the past decade highlights the extensive research interest in this material. One area that has a great potential for improvement through the integration of MXenes is sensor design. Strain sensors, temperature sensors, pressure sensors, biosensors (both optical and electrochemical), gas sensors, and environmental pollution sensors targeted at volatile organic compounds (VOCs) could all gain numerous improvements from the inclusion of MXenes. This report delves into the current research landscape, exploring the advancements in MXene-based chemo-sensor technologies and examining potential future applications across diverse sensor types.

## 1. Introduction

**Figure 1:**
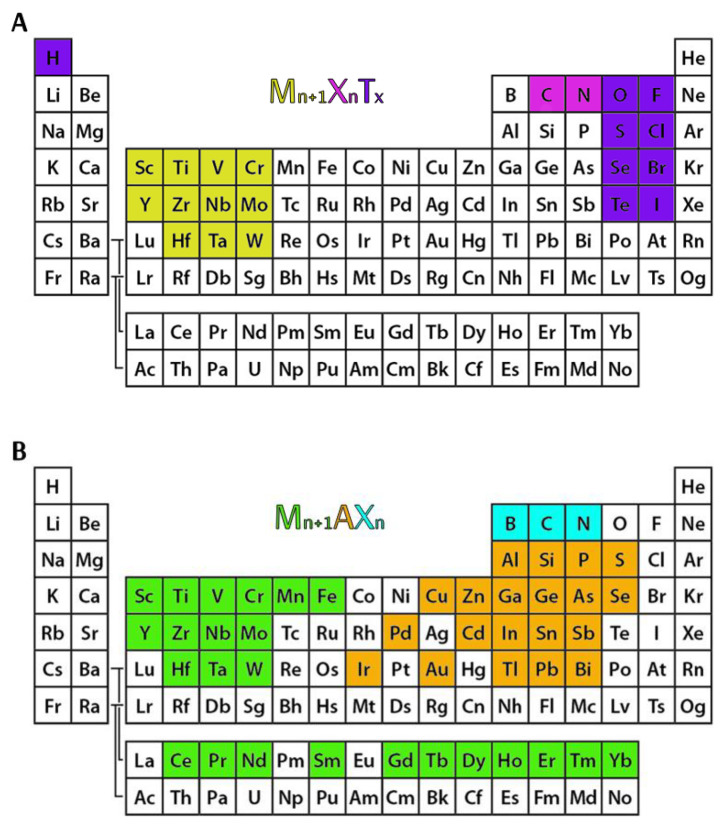
Elements of the known MXenes (**A**) and MAX phases (**B**).

The family of two-dimensional (2D) transition metal carbides, nitrides, and carbonitrides called MXenes has significantly grown since the first report of the synthesis of Ti_3_C_2_ in 2011 [1]. In recent years, MXenes have greatly impacted many fields, including energy storage [2,3], electronics [4,5], biomedicine, catalysis [6,7], environmental technologies, and many other technological areas. This widespread attention arises from the exceptional set of properties attributed to MXenes, which include superior mechanical strength and flexibility [8], liquid-phase processability [9], tunable surface functionality [10], high electrical conductivity [11], and the ability to customize their properties. This array of highly functional and desirable material qualities inevitably led to the production of thousands of publications during the previous ten years [12]. MXenes are characterized by the general chemical formula M_n+1_X_n_T_x_, where M is one of the transition metals, n varies from 1 to 4 [13], X indicates C and/or N, and T_x_ denotes the surface terminations, which include –F, –O, and –OH as well as other chalcogens (–S, –Se, –Te), halogens (–Cl, –Br, –I), or imido (=NH) groups [14,15]. The suffix “ene” underscores their structural similarity to graphene and to other 2D materials [16]. Figure 1 shows the elements of the known MAX phases and MXenes.

One of the many reasons why MXenes are used in a variety of industries is the diverse chemistry associated with the element “M”. For instance, Vanadium (V)-based MXenes have a low ion diffusion barrier and some characteristics required for energy storage devices [17], Molybdenum (Mo)-based MXenes have great potential for application in the fields of electrocatalysis and thermoelectricity [18], and Niobium (Nb)-based MXenes exhibit magnetic phase change due to their diamagnetic nature [19]. However, Ti_3_C_2_T_x_ remains the most studied and commonly used MXene.

**Figure 2:**
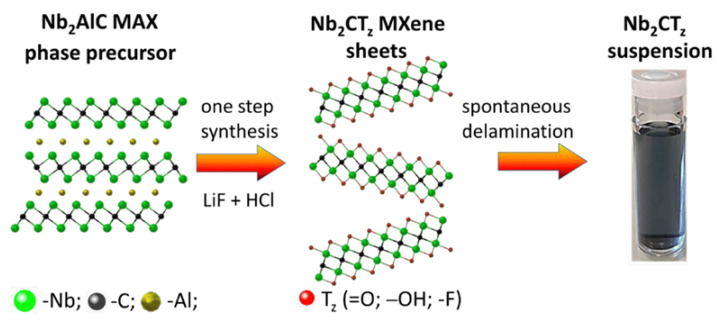
Schematic depiction of Nb_2_CT_z_ MXene synthesis. Reprinted from [39].

Etching the “A” element from the MAX phase, which is represented by the formula M_n+1_AX_n_, is a common step in the MXene synthesis process [20,21]. HF etching is the most popular etching technique, which is applied for the preparation of MXenes from a particular MAX phase [22,23,24,25]. However, the primary issue with this approach is the corrosive and hazardous nature of HF, which raises concerns about potential harm to the environment and public health. To overcome this issue, scientists have recently developed a number of innovative etching methods. Alkali etching [26,27], molten salt etching [28,29], electrochemical etching [30,31], iodine etching [32] and UV-induced etching [33] are some of the many new approaches for synthesizing MXenes. After the etching, multilayered MXenes are created and can already be utilized in some applications (such as lubrication [34]). However, in many other cases, MXenes must be intercalated and delaminated in order to optimize the chemical characteristics of these materials. DMSO [35], bases such TBAOH [36] and isopropylamine [37], and a variety of cations (Li^+^ [13], Na^+^ [38], K^+^ and others [23]) are used as intercalants during the delamination of MXphase and the formation of 2D MXene sheets. Figure 2 shows a schematic depiction of Nb_2_CT_z_ MXene synthesis, depicting all major steps necessary for MXene formation.

MAX phases are usually produced by combining laboratory-grade elemental powders (Ti, Al, and C powders for Ti_3_AlC_2_) and heating the mixture in an inert atmosphere to extreme temperatures; as such, this makes the production of the precursor relatively expensive compared to other two-dimensional substances (in comparison to obtaining graphene from graphite). Consequently, MXene production is comparatively expensive [40]. As a result, the developments in some areas may be hindered as it becomes economically unfeasible. Some research groups were able to synthesize MAX phases using inexpensive components (such as TiO_2_, Al from scrap metal, and C recovered from tires) and then utilize the obtained product to create MXenes. The comparison of properties between MXenes made from pure MAX phases and the ones from the inexpensive precursors exhibited similarities that are sufficient for certain applications. This shows that much more affordable MXene synthesis can be achieved and successfully applied as well [40,41]. However, for many other fields, especially ones that require highly pure MXenes, further improvements in synthesis are still needed.

MXenes are among the greatest materials that are used for sensing applications due to their various properties and the ability to tune them for users’ purposes by changing “M”, “T_x_” or the number of layers [42]. The addition of MXenes to sensor designs can improve strain sensors, temperature sensors, pressure and gas sensors and chemical sensors. Therefore, the aim of this report is to examine the state of research and potential future applications of some MXene-based sensors. In this paper, we discuss the benefits that MXenes can provide to different types of sensors, examine the mechanisms or reactions behind the sensing, and highlight the most unique and interesting works of the past decade. The main focus will be placed on outlining fairly recent (2022–2023) and, in our opinion, the most exciting advancements in the aforementioned sensor types. Moreover, the review will focus not only on the most popular Ti_3_C_2_T_x_ sensors but also on other novel MXene applications. It must be noted that biosensing applications of MXenes will not be discussed due to the enormously wide scope of this particular topic.

## 2. MXene-Based Strain Sensors

**Figure 3:**
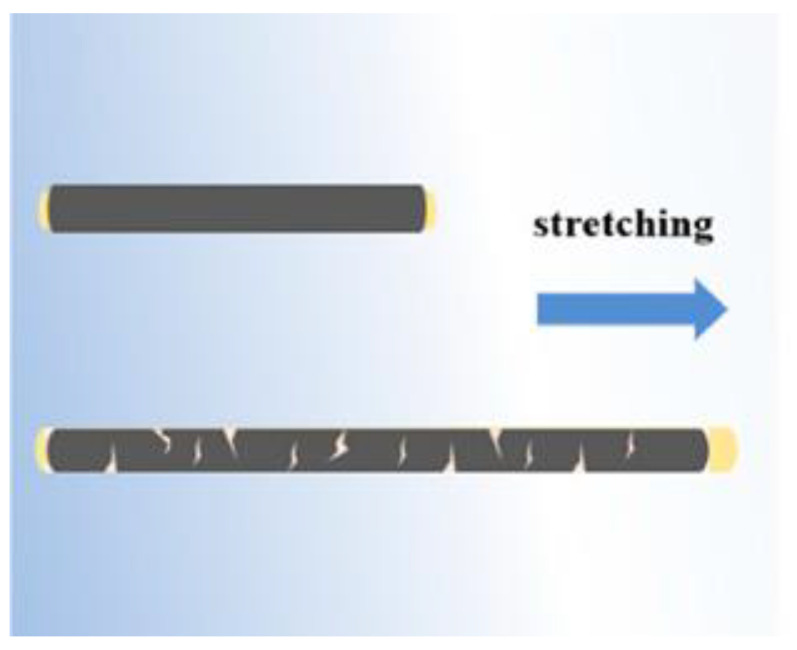
Schematic illustration of the crack propagation mechanism behind MXene-based strain sensors. Reprinted from [54].

The rapid advancement of flexible electronics-based devices in recent years has led to their widespread adoption across numerous industries. These devices, characterized by their bendable and conformable nature, have unlocked new possibilities and applications in fields such as sports, communications, medicine, and beyond. The versatility of flexible electronic devices has spurred innovation and opened new avenues for interdisciplinary research. As technology continues to advance, it is likely that flexible electronics will play an even more significant role in shaping the future of various industries [43]. For wearable applications like human motion detection, strain sensors need to meet several baseline requirements, including high stretchability, flexibility, sensitivity, and durability. To achieve this, scientists need to carefully design and coordinate different materials. One method is to insert zero-dimensional nanoparticles, such as metal nanoparticles [44,45], into an elastic substrate to create a conductive network, which can help in achieving high sensitivity. However, steady sensing may be hampered by the instability of the conductive network. On the other hand, one-dimensional nanowires like CNTs [46,47] and AgNWs [48] can be used to create a conductive network inside or on top of the elastic substrate. Because of the relatively stable conductive channels provided by this construction, resistance changes are less likely to occur due to deformation, yet this comes at a cost of decreased sensitivity [49]. Contrary to most other two-dimensional materials, such as graphene, MXenes have a large initial metallic conductivity. Since the crack propagation mechanism (Figure 3) dominates the sensing, MXene-based strain sensors exhibit increased resistance during stretching [50]. A narrow sensing range is the result of MXene sheets’ tendency to lose inter-sheet connections during the tensile process [51,52,53]. Combining conductive materials can enhance the overall conductivity and performance by taking advantage of different qualities of the materials.

One of the profound features of MXenes in strain sensor design is the ability to tune properties, such as the sensitivity and working area. Ti_3_C_2_T_x_ An MXene/carbon nanotube (CNT) composite was conceptually designed and included in strain sensors by Yichen Cai et al. CNT crossing and Ti_3_C_2_T_x_ nano-stacks were combined in a weaving architecture. The resulting strain sensor had an ultralow detection limit of 0.1% strain, tunable sensitivity (gauge factor up to 772.6), and customizable sensing range, mostly depending on the concentration ratio between CNT and MXene (30% to 130% strain) [52]. Wireless Ti_3_C_2_T_x_ MXene strain sensing devices were created by Haitao Yang et al. Within several user-specified high-strain working windows (from 130% to 900%), the wireless MXene sensor system may simultaneously achieve an ultrahigh sensitivity (with gauge factor up to 14,000) and reliable linearity (R^2^ = 0.99). The authors used a single database channel by the wireless system to track all of the exoskeleton actuations [55].

**Figure 4:**
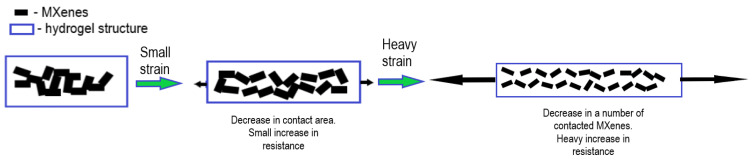
Schematic illustration of the mechanism behind MXene-based hydrogel strain sensors.

Due to the hydrophilicity of MXenes, based on surface terminations, they can be implemented in hydrogels. Figure 4 shows a schematic illustration of the mechanism of MXene-based hydrogel strain sensors. Hui Liao et al. immersed MXene nanocomposite hydrogel (MNH) into ethylene glycol to create an anti-freezing, self-healing, and conductive MNOH. According to the authors, MNOH may be incorporated into a wearable strain sensor with a reasonably wide strain range (up to 350% strain) and a high gauge factor of 44.85 under very low temperatures to monitor human biological activity [56].

In 2023, Chunqing Yang et al. created a sandwich-structured flexible strain sensor using MXene, polypyrrole, hydroxyethyl cellulose (MXene/PPy/HEC), and a flexible substrate made of PDMS. The signals of handwritten Chinese, Arabic, and English words measured by the sensor exhibited distinctive properties. Different handwritten characters were successfully recognized by the authors using machine learning techniques, with a recognition accuracy above 96% [57].

In conclusion, MXenes, especially Ti_3_C_2_T_x_, have been successfully applied in strain sensor design due to their superior electrochemical properties, metallic conductivity, and hydrophilicity. They are able to provide high sensitivity to strain sensors at the cost of narrowing the working area. More examples of MXene usage in strain sensors can be found in Table 1.

## 3. MXene-Based Pressure Sensors

**Figure 5:**
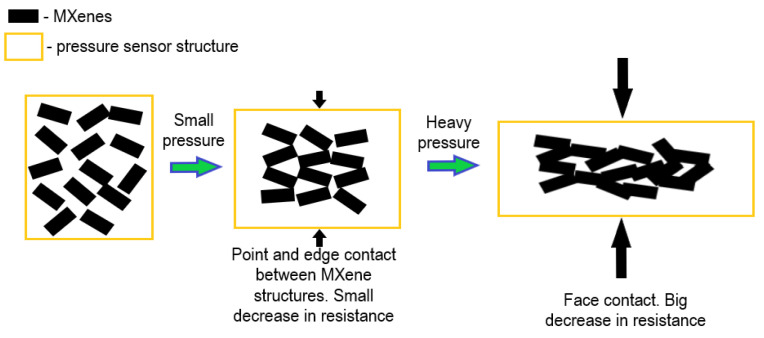
Schematic illustration of the mechanism behind MXene-based pressure sensors.

Flexible pressure sensors have become integral components in wearable electronic devices, offering a wide range of applications in areas such as health monitoring, human-machine interfaces, and robotics. In the past decade, numerous studies have focused on exploring various mechanisms employed in flexible pressure sensors, including piezoresistive, piezocapacitive, piezoelectric, and triboelectric principles. These mechanisms enable these sensors to detect and respond to pressure changes in different ways. The Piezoresistive mechanism involves changes in the electrical resistance as a response to mechanical deformation. Flexible pressure sensors using piezoresistive materials, such as certain polymers or carbon-based composites, can detect pressure by measuring variations in resistance [69]. The Piezocapacitive mechanism is mostly based on monitoring the capacitance changes, which are generated in response to pressure and are the basis of piezocapacitive sensors. These sensors typically consist of flexible materials with varying capacitance, and the pressure-induced deformations lead to changes in the capacitance that can be measured [70]. The Piezoelectric mechanism is realized in materials with piezoelectric properties to generate an electric charge in response to mechanical stress. Flexible pressure sensors based on the piezoelectric mechanism use such materials to convert pressure-induced deformations into electrical signals [71]. Triboelectric sensors generate electric charges through the triboelectric effect, which involves the transfer of electrons between materials in contact. Flexible pressure sensors based on triboelectric principles can detect pressure changes through the generation of triboelectric charges [72]. All these mechanisms offer different advantages and are suitable for various applications based on the specific requirements of the wearable device. The choice of the mechanism depends on factors such as the required sensitivity, response time, and the overall design of the sensor. The ongoing research and development in this field aims to improve the performance, sensitivity, and flexibility of these pressure sensors, contributing to the continued evolution of wearable electronic devices and their integration into diverse applications [73,74,75,76,77]. Piezoresistive pressure sensors stand out among the others due to the ease of device assembly and the comparatively low requirements for signal collection and readout [78]. The structure of all types of pressure sensors consists of flexible substrates, active materials, and conductive electrodes despite the fact that different sensing mechanisms are used. High sensitivity, a wide detection range, rapid response time, low detection limit, and good linearity are required for the perfect flexible pressure sensor [79,80,81]. However, because of the constrained sensing capabilities and intricate manufacturing process, the practical application of pressure sensors is still challenging and expensive. A brand-new frontier in the development of pressure sensors has been opened by the discovery of MXenes and especially Ti_3_C_2_T_x_-based ones. The surface of Ti_3_C_2_T_x_, in contrast to other 2D materials like graphene and black phosphorus, has a great number of attached exchangeable functional groups, which gives them exceptional water dispersion and plasticity. They may also be mixed with other materials to create a variety of multifunctional arrangements and micro-structures. Ti_3_C_2_T_x_ nanosheets are noteworthy due to their advantageous mechanical properties, high conductivity, and pressure-adjustable layer spacing. These features offer a solid foundation for the design of microstructures, which are required for pressure sensors, tactile sensors, and the formation of force-sensing layers [82]. Figure 5 shows the mechanism behind MXene-based pressure sensors.

An MXene/cotton fabric (MCF)-based pressure sensor was developed in 2020 by Yanjun Zheng et al., utilizing the flexibility and three-dimensional porous structure of cotton fabric as well as the unique sandwich construction of the sensor. It displayed advanced stability, long-term durability, high sensitivity (5.30 kPa^−1^ in the pressure range of 0–1.30 kPa), a wide sensing range (0–160 kPa), and a quick response/recovery time (50 ms/20 ms) [83]. A flexible, extremely sensitive, and waterproof sponge pressure sensor was described by Zhenyuan Xu et al. SiO_2_, MXene, and NH_2_-CNTs were put together to create the sensor on a melamine sponge core. Good sensitivity (10.8 kPa^−1^), a quick response and recovery time (40 ms and 60 ms, respectively), a broad detection range (30 kPa), and a remarkably low detection limit (4.6 Pa) were all features of this waterproof sponge pressure sensor [84]. Lin Wang et al. created an MXene/ANFs composite aerogel through a controlled vacuum filtering procedure followed by freeze-drying. The aerogel sensor exhibited a broad detection range (2.0–80.0% compression strain), good sensitivity (128 kPa^−1^), and a low detection limit (100 Pa) [85]. In 2022, Tingting Yin et al. developed MXene/polyaniline (PANI) foam utilizing a steam-induced foaming technique to produce a 3D porous structure. This foam-based sensor demonstrated exceptional wear resistance (10,000 cycles), quick response and recovery times (106/95 ms), and remarkable sensitivity (690.91 kPa^−1^) [86]. In 2022, using electrospinning technology, Xiyao Fu et al. created a pressure sensor using the MXene/ZIF-67/polyacrylonitrile (PAN) nanofiber film. The film-based device exhibited a broad operating range (0–100 kPa), good sensitivity (62.8 kPa^−1^), robust mechanical stability (over 10,000 cycles), and a quick response/recovery time (10/8 ms) [81].

Sound, which is essentially a ‘vibration’ of molecules, may travel through a gaseous/liquid/solid medium and can be transmitted as mechanical waves based on the variation of pressure and motion [87]. Exploring communication systems that use other modes of signals instead of sound is important as various human infirmities can restrict or impede the capacity to talk [88]. Compared to layered graphene or other materials, MXene nanoflakes with considerably varied interlayer spacing may produce a higher mechanical response, making them an appropriate mechanical sensing material for developing piezoresistive devices for sound registration [89]. Yangyang Pei et al. used foamed MXenes for their sound sensor. This method enabled this group of scientists to tackle the MXenes’ self-stacking problem, a common limitation that restricts material applications, by reducing the total energy. The Ti_3_C_2_T_x_-based sensor demonstrated exceptional durability over 5000 cycles, a low detection limit of 1 Pa, a quick reaction time of 132 ms, and an impressive sensitivity of 102.89 kPa^−1^ for pressures less than 1 kPa. The sensor demonstrated the ability to perceive air pressure waves as an eardrum, distinguishing between diverse natural sounds and identifying human voices [90]. Guang-Yang Gou and others reported on an ultrasensitive intelligent artificial eardrum based on MXene and PDMS-PE. This artificial eardrum demonstrated an unprecedented sensitivity of 62 kPa^−1^ and a very low detection limit of 0.1 Pa. The accompanying machine-learning algorithm for real-time voice classification developed by the authors showcases a high accuracy of recognition of 96.4 and 95% [91].

The aforementioned examples of MXenes in pressure sensing applications highlight how its unique properties contribute to significant advancement in this field. High conductivity, hydrophilicity, abundance of changeable surface functional groups, customizable layer spacing and several other key characteristics of MXenes make this material particularly well-suited for pressure sensor development [92]. Most importantly, MXenes typically exhibit high electrical conductivity, which is essential for efficient signal transmission in pressure sensors. This property allows for accurate and rapid detection of pressure changes [69]. Moreover, MXenes are hydrophilic, meaning they have a strong affinity for water molecules. This property can be advantageous in pressure sensors designed to operate in humid or wet conditions as it helps maintain sensor performance in such environments [84]. MXenes’ surfaces contain an abundance of changeable functional surface groups that can be modified or altered. This provides researchers and engineers with the flexibility to tailor the surface chemistry of MXene-based pressure sensors, optimizing their sensitivity and selectivity to specific stimuli [93]. The layered structure of MXenes allows for the adjustment of interlayer spacings. Customizable layer-spacing-based tunability is valuable in pressure sensors as it enables customization to enhance the sensitivity and responsiveness to pressure variations [94].

Aerogels, hydrogels, and foams with a highly organized porous microstructure are included in the force-sensitive layers, which helps to increase the sensitivity and pressure response range. The pressure sensor’s response time and sensitivity can be increased by improving the contact between the force-sensitive layer and the electrode layer in fiber composites and fabrics made from MXenes. As a result, there is a lot of development potential for pressure sensors based on MXenes. More examples of MXenes used in pressure sensors are presented in Table 2.

In conclusion, MXenes already play a part in the current advancements in pressure sensing technologies as they exhibit highly desired and useful qualities for sensor design including high conductivity, hydrophilicity, abundance of changeable surface functional groups, and customizable layer spacing. These inherent characteristics of MXenes contribute to its versatility in pressure sensing applications, making it a promising material for further development of advanced and high-performance pressure sensors. The inclusion of MXenes could benefit a diverse range of pressure sensors including those used in touchscreens, wearable devices, and structural health monitoring. Ongoing research is likely to further explore and optimize MXene-based pressure sensors for enhanced performance and expanded applications.

## 4. MXene-Based Temperature Sensors

**Figure 6:**
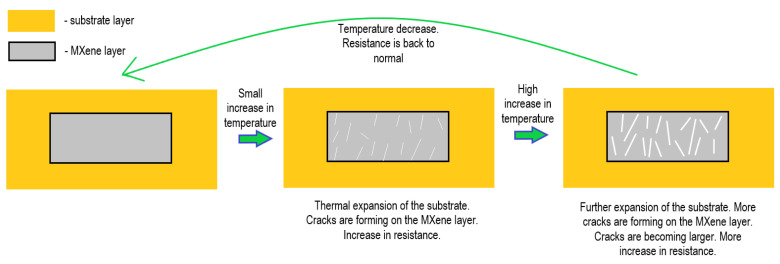
Crack mechanism of temperature sensors with MXenes.

Temperature sensors are used in various industries: from kitchen appliances to patient monitoring and reaction temperature monitoring. The sensing abilities of most temperature sensors are based on the variation in electrical properties of materials, which are sensitive to temperature. The sensing mechanisms behind MXene-based temperature and strain sensors are quite similar and are dominated by the so-called ‘crack mechanism’. Such temperature sensors consist of a temperature-sensitive substrate that expands with increasing temperature with an MXene-based layer deposited on the top. When the temperature rises, the thermal expansion of temperature-sensitive material places tensile stress on the MXene-based layer, causing it to displace and ‘crack’, which results in an increase in electrical resistance; the resistance gradually returns to its initial value when the temperature-sensitive substrate shrinks (Figure 6) [100].

Lianjia Zhao et al. developed a flexible temperature sensor using sodium alginate hydrogel as a heat-sensitive coating layer and Ti_3_C_2_T_x_ MXene as a conducting skeleton. With a sensitivity up to 3244% °C^−1^, the sensor displays an accurate and consistent temperature response over the whole range (from −20 to 100 °C). The sensitivity of the obtained sensor was higher compared to a metal oxide sensor, CNT sensor and PEDOT:PSS/GO sensor. It is worth noting that the linear range of the sensor was lower compared to that of the CNT sensor [101]. Zeyi Wang et al. created a Fe^2+^/Ti_2_CT_x_/CA-PAM hydrogel-based frost- and dehydration-resistant temperature sensor. The sensor demonstrated good linearity (R^2^ = 0.998), sensitivity (−1.07% °C^−1^) across a wide operating range (−10 to 60 °C), high resolution (0.1 °C), and good repeatability. Compared to most other fire detection and warning sensor systems (based on nanoparticles, graphene oxide and metal oxides), the developed sensor demonstrated a greater reproducibility of fire detection and the benefit of the absence of a need for external power to function. The sensor’s potential in healthcare monitoring, electronic skins, and intelligent robotics was also demonstrated by its integration into a wireless system for body temperature monitoring [102]. By dip-coating Ti_3_C_2_T_x_ MXene material on a polyester fiber substrate and depositing silver nanoparticles, Hailian Liu et al. were able to create a high-performance wearable sensor. The fiber sensor had great temperature sensitivity (1.0436% °C^−1^–3.4938% °C^−1^), in addition to responding well to stimuli of temperature, pressure/strain signals [103]. Ti_3_C_2_T_x_ nanoparticle–lamella hybrid networks were incorporated into PDMS substrates by Zherui Cao et al. to create a flexible temperature sensor. The sensor had a high sensitivity (up to 986% °C^−1^) and a broad response range (up to 140 °C), quick response time—below 7.0 s, high accuracy (0.1 °C), and good dependability and durability of >100 cycles, which could meet the needs of e-skin for temperature monitoring [100]. Using graphene and Ti_3_C_2_T_x_ MXene nanoinks, Mortaza Saeidi-Javash et al. presented an aerosol-based flexible bimodal sensor. The printed temperature sensor exhibited a competitive ’thermo-power’ output of 53.6 V/°C with extremely high precision and stability. It should be noted that even after 1000 bend cycles, the printed MXene-based sensor still exhibited outstanding flexibility with barely any degradations [104].

**Figure 7:**
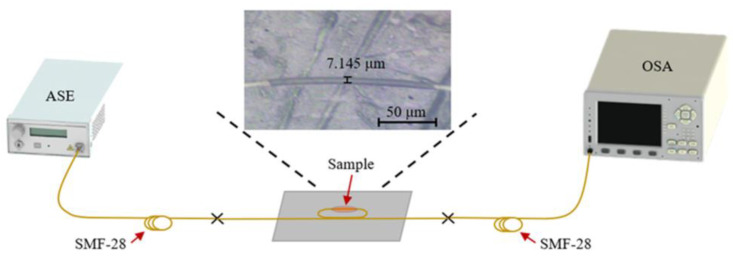
Experimental setup for temperature sensing. Reprinted from [106].

To create a photothermal optical sensor (PHOS), Yan Zuo et al. added Ti_3_C_2_T_x_ into the Mach–Zehnder interferometer (MZI) framework. An efficiency as high as 0.19 π·mW^−1^·mm^−1^ under 980 nm laser pumping was attained as a result of the Ti_3_C_2_T_x_’s effective photothermal conversion, and an even higher efficiency was seen when exposed to red light. The designed scheme’s response time was measured to be 23.4 s [105]. Si Chen et al. have demonstrated an all-fiber temperature sensor based on MXene V_2_C. When the temperature range was ~25–70 °C, the corresponding transmission light intensity variation was linear, with a maximum normalized sensing efficiency of 2.21 dB·°C^−1^·mm^−1^. Figure 7 shows experimental setup for measuring temperature, used in the latter experiment. The runway structure used in the sensor device significantly enhanced the interaction length between light and the V_2_C, thereby improving the overall sensing efficiency of the MXene-based sensor. Compared to other fiber sensors based on graphene or metal oxides, this sensor has superior sensitivity but a reduced linear range [106]. An MXene V_2_C integrated runway-type micro-fiber knot resonator (MKR)-based temperature sensor was presented by Qing Wu et al. in 2023. A maximum normalized temperature sensing efficiency of 1.65 dB °C^−1^·mm^−1^ was attained. The highest sensing efficiency (~0.33 dB·°C^−1^) was 2.7 times greater than the naked runway-type MKR’s (~0.09 dB·°C^−1^) [107].

MXene-based temperature sensors encounter specific challenges that warrant attention. Firstly, despite exhibiting superior qualities compared to some alternative materials, MXene-based sensors face accuracy issues when utilized for temperature measurements. Secondly, the current manufacturing methods for temperature sensors, which often involve distinct processing steps and intricate fabrication designs, may compromise the comfort of wearable devices. Overcoming these challenges is crucial for optimizing the performance and applicability of MXene-based temperature sensors in various applications [108]. In general, compared to other materials, MXenes usually provide advanced sensitivity but lower stability and smaller linear range.

## 5. MXene-Based Humidity Sensors

Monitoring and regulating humidity have become increasingly crucial in various industries, such as agriculture, textile technology, and food storage. The significance of controlling humidity levels stems from its direct impact on product quality, preservation, and overall operational efficiency within these sectors. As a result, precise humidity management is now integral to maintaining optimal conditions for crop growth, ensuring the quality of textiles, and preserving the freshness and safety of food products during storage and transportation. The evolving importance of humidity control underscores its pivotal role in enhancing performance and outcomes across diverse industrial applications [109,110,111]. For these applications, it is crucial to detect relative humidity (RH) with good accuracy, sensitivity, and reliability. Ti_3_C_2_T_x_ MXene possesses terminal groups that provide ample hydrophilic active sites for water adsorption and intercalation [112,113], in contrast to semiconductor metal oxides where water-molecule-dependent surface conductivity dominates the sensing mechanism [114,115]. The low electrical conductivity of water and the lengthening of the layer-to-layer distance will result in an increase in Ti_3_C_2_T_x_ resistance following the intercalation of water molecules in the interlayers between 2D MXene sheets [113,116]. Thus, Ti_3_C_2_T_x_ MXene provides a sensing mechanism that is suitable for RH sensing [117].

Layer-by-layer assembly was used by Hyosung An et al. to create MXene/polyelectrolyte (poly(diallyldimethylammonium chloride)) multilayer films, which displayed extremely quick response (110 ms) and recovery (220 ms) times in the RH range of 20–40%. The sensor was successfully employed to monitor human respiration in real time, though the authors noted that direct contact with the electrolyte can lead to skin irritation. It was also shown how increasing the space between sheets increased the tunable resistance by intercalating water molecules into multilayers [118]. Yang Lu et al. described the fabrication of an impedance-type humidity sensor based on Ti_3_C_2_T_x_/g-C_3_N_4_ nanomaterials. They investigated the process of humidity sensing using complex impedance spectroscopy, finding that the major conductive ion is H_3_O^+^ at RH between 11% and 43%. Physisorbed water layers began to grow on top of the initial chemisorbed water layer as the Grotthuss chain reaction (H_2_O + H_3_O^+^ → H_3_O^+^ + H_2_O) governed charge transportation at this stage, where the major charge carriers H^+^ move freely. As a result, the impendence dropped as the humidity increased, which formed the groundwork for the created sensor. At a range of 11–97% RH, the humidity sensor demonstrated exceptional repeatability, fast response and recovery times (4.8 and 8.9 s, respectively), and neglectable hysteresis. The response was significantly stronger than that of most other impedimetric sensors based on other materials (namely, Ag or LiCl). The authors showed how it might be used to detect water evaporation and monitor human breath [119]. Mimi Han et al. assembled MXene nanosheets into a layered TOCNF/MXene nanocomposite film utilizing vacuum-assisted filtration and (TEMPO)-oxidized cellulose nanofibers (TOCNFs) as a template. The swelling and contraction of channels between MXene interlayers caused by adsorbed H_2_O and the swelling of TOCNFs were both components of the sensor’s humidity-sensing mechanism. The TOCNF/MXene sensor displayed exceptional bending and folding durability (up to 50 cycles), long-term stability, and a maximum response (−∆I/I_0_) of 90% under 97% RH. The authors demonstrated the sensor applications in smart wearable electronics by dynamically monitoring human breath, skin, and fingertip humidity [120].

By fluoride doping, Runlong Li et al. improved the MXene quartz crystal microbalance-based humidity sensor. They demonstrated that the resulting sensor had 12.8 Hz/% RH sensitivity compared to 10.2% for the fluoride-free sensor that was made using the same procedure. The fluoride-doped sensor showed a rapid response time of 6 s and 2 s, a maximum humidity hysteresis of 1.16% RH, high short-term repeatability, long-term stability, and excellent selectivity in the range of 11.3–97.3% RH [121]. In 2017, Eric S. Muckley and colleagues developed a material for humidity detection by intercalating K and Mg between the MXene layers. They were able to demonstrate that the d-spacing was initially larger for Ti_3_C_2_-K (11.5Å versus 10.1 Å in Ti_3_C_2_-Mg) using neutron scattering. After hydration, Ti_3_C_2_-Mg had a greater d-spacing (14.6 Å against 12.4 Å in Ti_3_C_2_-K) as the smaller size and higher charge of Mg^2+^ allow for a stronger interaction with H_2_O, resulting in a larger hydrated radius of Mg^2+^ (~4.28 Å versus ~3.31 Å). The greater radius causes a bigger hydrated d-spacing in Ti_3_C_2_-Mg after hydration. The emerged Mg(H_2_O)_6_ cluster was shown to be the best candidate for the pillaring effect as a result of creating a stiff structure. Relative humidity (RH) detection thresholds of ~0.8% RH were found in Ti_3_C_2_-K and Ti_3_C_2_-Mg films, and they also demonstrated a monotonic RH response in the 0–85% RH range with ~3% resolution [122].

One of the other methods of improving sensitivity is alkaline treatment. Organ-like alkalized Ti_3_C_2_T_x_ sensors were created by Zijie Yang et al. to monitor NH_3_ and humidity at room temperature. The device based on alkalized Ti_3_C_2_T_x_ had opposite response signals with improved NH_3_ and humidity detecting capabilities compared to not alkalized Ti_3_C_2_T_x_. The Ti_3_C_2_T_x_ altered carrier type of conductivity following oxygen functionalization was the cause of the response signal’s change in direction [16].

## 6. MXene-Based Gas Sensors

The fast industrialization of society has created numerous issues related to volatile pollutants and greenhouse gases, posing threats to both the environment and human health. Detecting and quantifying the presence of harmful gases in a timely and precise manner are essential both for mitigating the negative effects of the pollutants and for providing accurate data for environmental research. MXenes are currently attracting significant attention in the field of gas sensing due to their exceptional qualities, such as their high metallic conductivity, abundant and tunable functional groups (-OH, -O, or -F), porous structure, and quick electron transfer ability [123,124].

**Figure 8:**
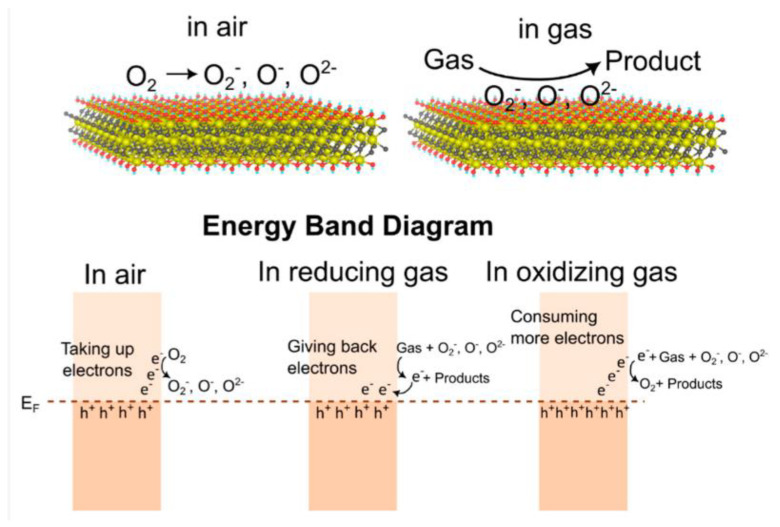
Gas sensing mechanism of the pristine 2D MXenes and the corresponding energy band diagram. Reprinted from [126].

The fundamental gas sensing process of pure MXenes’ is based on the adsorption and desorption of gas from the sensing layer and the ensuing change in the concentration of free charge carriers [125]. The alteration in the electrical conductivity of MXenes arises from the reaction of target gases (as demonstrated in Figure 8 for NH_3_) with surface defects and termination groups:

2NH_3_ + 3O^−^ → N_2_ + 3H_2_O + 3e^−^ (1)

NH_3_ + OH^−^ → NH_2_ + H_2_O + e^−^ (2)

Figure 8 show General gas sensing mechanism of pristine MXenes for NH_3_.

Despite their unique properties, pristine bare MXenes face certain limitations that hinder their effectiveness as sensing materials sensitive towards a broad range of gases. Challenges during the development of gas sensors include a lack of specificity to certain gas groups (excluding hydrogen-binding gases like acetone and NH_3_ as research shows MXenes have high selectivity towards it, compared to most other 2D materials [127,128]) and molecular structures, a relatively low bandgap, and a mechanical stability that is not always sufficient. These limitations can impact the sensitivity and selectivity of MXene-based gas sensors, making them less suitable for comprehensive gas sensing applications. Researchers and engineers are actively exploring various strategies to overcome these challenges. Surface functionalization, hybridization with other materials, and structural modifications are some of the approaches being investigated to enhance the performance and detection spectrum of MXene-based gas sensors [129,130]. Synergistic approaches with other materials, such as metal oxides, conducting or non-conducting polymers, 2D carbon nanomaterials, chalcogenides, and other surface functionalizing materials, might improve the properties of pure MXene sensors [16,122]. Depending on the characteristics of the components, the synergistic and reverse enhancing effect in MXene composites directly affects the sensing mechanism. For example, the basic mechanism for MXene composites containing metal oxides is quite similar to that of conventional metal oxide semiconductor-based sensors, in which the gas analyte’s effective absorption or desorption on the surface of the sensing material results in a change in the device’s resistance. The interfacial interactions between the two contributing materials and heterojunction production are related to the gas-sensing process of MXene-metal oxide composites [131].

### 6.1. NH_3_ Sensing by MXene-Based Sensors

Ammonia (NH_3_) is an important raw material in many domains. However, NH_3_ is a hazardous, corrosive, and colorless odor gas, and even at low concentrations can be dangerous to human health [132,133,134]. Therefore, quick and accurate monitoring of NH_3_ gas is crucial to ensure safety in industrial settings [135]. Xiao and co-workers revealed the advantages of using MXenes for NH_3_ sensing using first-principle simulation. The results showed that NH_3_ has a strong affinity to MXene substrates [136].

Seon Joon Kim and colleagues used interfacial self-assembly to create ultrathin Ti_3_C_2_ MXene films. The film was able to detect a concentration of 5 ppm NH_3_, and its sensitivity was 0.46% [137]. Eunji Lee et al. assembled Ti_3_C_2_T_x_ MXene sheets on flexible polyimide platforms using a straightforward solution-based technique. The constructed sensors responded strongly to ammonia (100 ppm with a sensitivity of 0.21%) [127]. Meng Wu et al. created Ti_3_C_2_ MXene-based sensors that had a varying-resistance-based response to numerous gases, namely CH_4_, H_2_S, methanol, ethanol, acetone, NH_3_, and NO. The sensors had good selectivity for NH_3_, with a 6.13% response, LOD of 10 ppm, and good linearity in the 10–700 ppm range [128].

Despite promising results, low sensitivity, long recovery time, and resistance drift after exposure to NH_3_ are still major issues for room temperature sensing of NH_3_ using pristine Ti_3_C_2_T_x_ MXene sensors [16,127,128]. Additional nanomaterials like graphene, carbon nanotubes (CNTs), reduced graphene oxide (rGO), and graphene oxide (GO) can be applied to promote the reaction with NH_3_ gas molecule [138]. By using a scalable wet-spinning technique, Sang Hoon Lee et al. created Ti_3_C_2_T_x_ MXene/graphene hybrid fibers. Excellent mechanical and electrical qualities made these fibers ideal for use in flexible wearable gas sensors. According to the authors, in comparison to pure MXene and graphene, the NH_3_ sensing response was considerably improved (∆R/R_0_ = 6.77%) in the created sensor [129].

Metal oxides are frequently chosen as sensing film materials for various sensing devices [139,140]. Metal oxides are also applied to further improve the NH_3_ sensing properties of resistive-based gas sensors because they can prepare a high amount of charge carriers [138]. TiO_2_- [141], nonstecheometric-TiO_2_- [142,143] and WO_3_-based [144,145,146] gas sensors are very promising for NH_3_ detection. In_2_O_3_/Ti_3_C_2_T_x_ MXene composites were created by Miao Liu et al. and displayed exceptional gas sensing qualities, including a high response (60.6%) to 5 ppm NH_3_ at RT, rapid response/recovery time (3/2 s), excellent selectivity, linearity, and good stability in the 5–100 ppm range. Compared to pristine MXene sensors and other metal oxides sensors, the designed sensor had better sensitivity and higher response to NH_3_ [147]. A hybrid heterojunction structure was created by Miao Liu et al. using the α-Fe_2_O_3_/Ti_3_C_2_T_x_ MXene composite material, which has a high specific surface area and a lot of functional groups. For 5 ppm NH_3_, the Fe_2_O_3_/Ti_3_C_2_T_x_ MXene sensor showed a response value of 18.3% and quick response/recovery times sub 2.5 s [135]. A ZnO/MXene hybrid surface acoustic wave (SAW) sensor was designed by Kedhareswara Sairam Pasupuleti et al. The sensor increased selectivity with an ultralow detection limit (89.41 ppb), displayed quick response/recovery time (92/104 s), long-term stability, and strong sensitivity under UV activation [148]. To improve the NH_3_ sensing capabilities of α-Fe_2_O, Miao Liu et al. created a hybrid structure of three components: Au, α-Fe_2_O_3_, and Ti_3_C_2_T_x_ MXene. The Au/α-Fe_2_O_3_/Ti_3_C_2_T_x_ MXene sensor displayed an excellent NH_3_ sensing performance, and its response value to NH_3_ (1 ppm) reached 16.9% at RT. Compared to other sensors (based on pure MXene or metal oxides), the obtained sensor had better sensitivity and response/recovery times [149].

The need for wearable NH_3_ sensors capable of real-time monitoring on diverse surfaces, including skin and clothing, is rather urgent. Polymer-based sensors have gained attention as one of the most promising options for addressing this requirement [150]. Polymer materials offer versatility and compatibility with a variety of substrates [151], making them well-suited for wearable sensor applications [152,153]. Polymer-based sensors can be designed to be flexible, lightweight, and adaptable, allowing for comfortable integration into wearable devices [140,154]. Additionally, polymers can exhibit sensitivity to specific gases, including NH_3_, and their properties can be tailored to enhance selectivity and performance [155,156]. The development of wearable NH_3_ sensors using polymer materials holds great potential for applications in various fields, including agriculture, environmental monitoring, and personal health. Ongoing research aims to optimize the design and performance of these sensors, paving the way for effective and versatile real-time monitoring solutions [157]. PEDOT:PSS/MXene composites were made by Ling Jin et al. and used in the fabrication of a dip-coated gas sensor. The composite sensor demonstrated a gas response of 36.6% against 100 ppm of NH_3_, with the response and recovery times being 116 s and 40 s, respectively. Additionally, it showed superior NH_3_ sensing performance at RT compared to pure PEDOT:PSS- and MXene-based sensors [158]. A portable self-powered NH_3_ sensor device was designed by Xingwei Wang et al. by implementing a bi-functional material made of 2D PANI/V_2_C MXene nanosheets. The authors discovered that the PANI/V_2_C nanosheets perform much better than pure PANI and MXene nanosheets in terms of response value (∆R/R_0_ = 14.9%), stability, and response/recovery time (9 s and 9 s) [159]. Zr_3_C_2_O_2_-based MXene is also a suitable material for room temperature NH_3_ sensors with excellent sensitivity and great selectivity, according to Xiumei Li et al. They proposed that a monolayer of Zr_3_C_2_O_2_ can be used as an NH_3_ detector since the NH_3_ molecule can be weakly chemically adsorbed with an adsorption energy of −0.597 eV, noticeable charge transfers (0.117 e), and a quick recovery time (1.05 ms) [160]. Such weak adsorption is a technological advantage because it enables easy and fast regeneration of the sensor. In conclusion, the discussed research results for MXene-based ammonia sensors showcase impressive applicability and potential for further development.

### 6.2. Determination of Volatile Organic Compounds (VOCs)

A wide range of sectors, including industrial safety control, environmental monitoring, and personal health management, have benefited greatly from the invention of gas sensors that are capable of accurately detecting volatile organic compounds (VOCs). These sensors play a crucial role in ensuring safety in industrial settings by monitoring the concentrations of VOCs and provide the necessary tools for environmental monitoring such as air quality evaluation. As VOC exposure can negatively impact personal health, sensors integrated into personal devices or wearable technology and able to provide real-time information about the surrounding air could ensure that the users are informed about the possible dangers of the environment they are in. Additionally, detection of VOCs emitted from the individual’s breath can also aid in early detection of certain illnesses and provide various health insights. Moreover, such sensors would be beneficial in residential and commercial spaces to monitor the indoor air quality and help in maintaining a comfortable and safe environment for the users of the space. Due to the abovementioned reasons, rapid advancements in VOC sensor technologies are important to our society as they contribute to improved safety, environmental sustainability, and overall human well-being in various areas [161,162,163]. Acetone sensors are gaining popularity due to their promising uses in self-diagnosis and health monitoring [164,165]. As an example, the acetone concentration in human breath is recognized as a crucial indicator for the identification of diabetes [166]. W_18_O_49_/Ti_3_C_2_T_x_ composites were made by Shibin Sun et al. by combining Ti_3_C_2_T_x_ MXene sheets with W_18_O_49_ nanorods. The obtained W_18_O_49_/Ti_3_C_2_T_x_-based acetone sensor exhibited an extremely low detection limit of 170 ppb, a quick response/recovery times (5.6/6 s to 170 ppb of acetone), and a high response of 11.6 to 20 ppm of acetone. The sensor also demonstrated nearly ideal selectivity towards acetone. However, the main problem with this parameter was the high response to ammonia, though authors claim that due to slower response and recovery rates to NH_3_, this should not be an issue. According to the authors, the distribution of W_18_O_49_ on the Ti_3_C_2_T_x_ surface, the removal of the fluorine terminational groups from the MXene, and the synergistic interactions between the W_18_O_49_ and the Ti_3_C_2_T_x_ provided the combined sensors superior acetone-sensing properties in comparison to W_18_O_49_ and Ti_3_C_2_T_x_ sensors [167]. The 3D Ti_3_C_2_T_x_ MXene/rGO/CuO aerogel sensor developed by Miao Liu et al. responded to 100 ppm acetone with a 52.09% rate at room temperature and demonstrated a quick response/recovery time (6.5 s/7.5 s) as well as high repeatability and selectivity. According to the scientists, the results are due to the highly interconnected porous structure and the combined effects of MXene, rGO, and CuO. Compared to pristine MXene and rGO/In_2_O_3_ sensors, the obtained sensor had a superior response/recovery time and higher response [168]. An urchin-like V_2_CT_x_/V_2_O_5_ MXene hybrid sensor was developed by Sanjit Manohar Majhi et al., and it demonstrated an increased response (S% = 11.9) toward 15 ppm acetone, a low limit of detection (250 ppb), and a quick response/recovery time (115s/180s). The selectivity test showed higher response to acetone in comparison to CO, H_2_, CO_2_, H_2_S and C_2_H_4_ (47% versus <15% towards other gases). According to the authors, the potential production of H-bonds in multilayer V_2_C MXenes, the synergistic effect of the created V_2_C/V_2_O_5_ composite, and the strong charge carrier transport at the V_2_O_5_ and V_2_C MXene interface can be applied for improving the sensing properties [169].

Gaseous toluene (C_6_H_5_CH_3_) is a dangerous VOC that is created during the process of tanning leather and other materials. Even at rather low concentrations, it is deadly to humans and very harmful to the environment [170,171]. To decrease the risk to one’s health from exposure to toluene gas, it is crucial to detect it early and monitor its concentration both indoors and outdoors. CuO/Ti_3_C_2_T_x_ MXene hybrids were created by Angga Hermawan et al., who also attributed the increased toluene gas sensing response of 11.4, selectivity, response (270 s) and recovery times (10 s) to the strong metallic phase conductivity of Ti_3_C_2_T_x_. However, the developed sensor had some significant issues with selectivity. The response signal to toluene was not much higher compared to ethanol and acetone signals (~11% versus 7%, respectively) [172]. A toluene sensor based on the MXene Mo_2_CT_x_ was proposed by Wenzhe Guo et al. It had an LOD of 220 ppb, an adequate selectivity for toluene against other VOCs (2.75% response compared to 1% of benzene and even lower responses towards other analytes), and a good sensitivity of 0.0366 Ω/ppm (140 ppm for toluene) [173].

Formaldehyde (HCHO) has been regarded as the primary contributor to sick building syndrome [174,175]. HCHO can cause the irritation of mucous membranes, skin, eyes and throat, cause shortness of breath, headaches, nausea, and possibly lead to cancer. This substance is one of the most harmful and carcinogenic VOC gases in urban and indoor environments [176,177,178]. Therefore, it has become necessary to create high-performance portable HCHO sensors. Co_3_O_4_ and ZnO/Ti_3_C_2_T_x_ MXene nanowire arrays were used to create the composite-based formaldehyde sensor that was proposed by Dongzhi Zhang and colleagues. The MXene/Co_3_O_4_ composite sensor demonstrated selectivity (~9.5% compared to the highest of ~1.75% towards acetone) and outstanding response characteristics (9.2 to 10 ppm HCHO, with an LOD of 0.01 ppm). The selectivity to HCHO was also improved compared to pure MXene and Co_3_O_4_ sensors. The synergistic interfacial interactions between MXene and Co_3_O_4_ were thought to be the cause of the improved responsiveness to HCHO [179]. To design a formaldehyde sensor, Gaoqiang Niu et al. applied a pre-oxidation technique to change SnO_2_/Ti_3_C_2_T_x_ MXene into SnO_2_/TiO_2_/Ti_3_C_2_T_x_ MXene nanocomposites. Due to the synergistic interaction between SnO_2_ nanosheets and TiO_2_/Ti_3_C_2_T_x_ MXene, the sensor demonstrated a high response rate (38.6 at 160 °C to 20 ppm with LOD being 50 ppb), strong selectivity (38.4 compared to the highest 16.0 towards ethanol), and reproducibility. When the selectivity of the sensor was compared to a pure SnO_2_ sensor, the results showed clear improvement of the signal (38.4 compared to <20%) [180].

Another VOC, methanol, is used as an organic solvent in numerous sectors, including the pharmaceutical, chemical, biomedical, and automotive industries. However, it is extremely combustible and has a low explosive limit of 6% in air. When a person is exposed to more than 200 ppm of methanol for 8 h, it can lead to intoxication [181]. Additionally, unlike other alcohols, methanol oxidizes slowly and builds up in the human body, which can result in poisoning. Therefore, it is crucial to design a methanol sensor that has good sensitivity and selectivity and can operate at ambient temperatures [182]. In_2_O_3_ nanocubes/Ti_3_C_2_T_x_ MXene nanocomposites showed strong responsiveness (29.6% to 5 ppm) and noticeable selectivity to methanol against other gases (30% towards methanol versus <18% towards trimethylamine, acetone, xylene and other gases) at room temperature, as reported by Miao Liu et al. According to the authors, the sensor was characterized by quick reaction and recovery times (6.5 and 3.5 s, respectively) to 5 ppm methanol at ambient temperature [183].

Ethanol is employed extensively in the pharmaceutical, food manufacturing, and biomedical industries as a key chemical ingredient [184,185]. Ethanol also poses a serious risk to the safety of production and transportation due to its combustible and explosive nature (explosion range of 3.3% to 19%) [186]. Additionally, long-term exposure to an atmosphere even with low levels of ethanol (25 ppm) can lead to serious negative health effects, including headaches, liver damage, and neurasthenia [187,188]. Therefore, designing and creating sensing materials for monitoring low-concentration ethanol gas at low temperatures is greatly import. Shuai Zhang et al. developed MoO_3_/Ti_3_C_2_T_x_ MXene nanocomposites employing a straightforward hydrothermal synthesis technique for efficient monitoring of low ethanol concentrations. The sensor responded well to low-concentration ethanol gas at 100 °C (R_a_/R_g_ = 1.61/1 ppm) and recovered quickly (10 s/49 s). Outstanding selectivity for ethanol was obtained, with the response being five times higher compared to other gases, like methanol and acetone [189]. In order to create resistance-type gas sensors, Chen Wang et al. produced SnO_2_/MXene composites that were brushed onto a MEMS platform. The composition significantly increased the sensitivity of SnO_2_-based gas sensor, with ethanol gas having the maximum sensitivity. The composite additionally accelerated the sensor’s response and recovery times (14s and 26s, respectively) and reached a 5.0 response value to 10 ppm ethanol at its ideal temperature of 230 °C, which was twice as high as the response value of a pure SnO_2_ sensor. Though the selectivity of the sensor was improved after adding MXenes, the response towards acetone is still comparable to ethanol (~4 towards acetone compared to ~5 towards ethanol). The improvement in the composite’s gas-sensing characteristics is attributed, according to the authors, to its Schottky barrier and the metal-like conductivity of Ti_3_C_2_T_x_ [190].

### 6.3. Other Gas Sensors Based on MXenes

There are other gases that can be monitored by MXene-based gas sensors. One of them is H_2_, a gas with a significant energy capacity that is renewable and sustainable. It is commonly used in the metallurgy, fuel cell, and automotive industries [191,192]. However, H_2_ gas is highly explosive in air at concentrations of more than 4% and has a low ignition energy. Additionally, due to its small molecule size and high diffusion coefficient, it leaks during usage, storage, and transit [193,194]. Because H_2_ gas is odorless, our sense of smell system is unable to detect it; hence, the creation of high-response H_2_ sensors is essential to guaranty safety. A unique Pt = Pd/Ti_3_C_2_T_x_ hydrogen sensor was developed by Lei Wang et al. using a simple hydrothermal chemical reduction process. The resulting sensor had a response of 24.6% (200 ppm H_2_ at RT), a response/recovery time of 6/8 s, an LOD of 200 ppm, and strong selectivity (24.6% for hydrogen compared to the highest towards ethanol 5.2%) in addition to good linearity, long-term stability, and repeatability [195].

The main sources of nitrogen dioxide (NO_2_) are industrial combustion, automotive exhaust, and coal mining. According to research, even very small concentrations of NO_2_ (~1 ppm) can harm the respiratory system and eyes to varying degrees. Furthermore, persistent exposure to higher NO_2_ concentrations may possibly be fatal to humans [196]. Fuqiang Guo et al. effectively created Ti_3_C_2_T_x_/CuO nanocomposites using an eco-friendly and straightforward hydrothermal process for NO_2_ sensing. The response of the author’s nanocomposite sensor to NO_2_ (56.99%) was five times greater than that of the pure Ti_3_C_2_T_x_ sensor. The improved gas sensing capability was attributed to higher oxygen vacancies and adsorbed oxygen in comparison to Ti_3_C_2_T_x_ as well as hybrid heterojunctions between CuO and Ti_3_C_2_T_x_. The Ti_3_C_2_T_x_/CuO sensor demonstrated extremely quick response and recovery times (16.6/31.3 s to 20 ppm NO_2_), exceptional selectivity to NO_2_ (with the response being ~57% towards NO_2_ versus 31.09% towards NO and 12.63% towards ammonia), and long-term stability [197].

Other researchers have demonstrated that other gases, such as NO_x_ [198,199], hexanal [200] and others [201,202], can also be determined by gas sensors based on MXenes. It can be predicted that due to the unique characteristics of MXenes, such as tunable terminal groups, and metallic conductivity, they have been successfully applied in gas sensor design. Compared to other materials, pristine MXenes can offer high sensitivity with mediocre selectivity (except for hydrogen bonding gases) and a lower linear range. Combining MXenes with other more selective or more stable materials (especially with metal oxides or metal nanoparticles) usually results in much better results.

## 7. Chemical Sensors

The socio-economic changes induced by industrialization in the middle of the 19th and 20th centuries have considerably improved the quality of life of many people. However, such advancements have also resulted in pollution and contamination of water, soil, air, etc. Naturally, MXene nanomaterials have drawn a lot of interest in the field of pesticide sensing, heavy metal sensing, etc., because of their exceptional electrical and optical properties. Efficient functionalization of MXenes is made possible by the surface termination groups, which is essential for electrochemical sensing [203] as they give MXenes a superb reducing ability. Qualities of MXenes make them suitable for improvement of the functionality of sensors and biosensors, providing higher sensitivity, selectivity to some analytes, and strong reproducibility but shorter detection ranges compared to other materials. Though MXenes have remarkable electrocatalytic activity, one of their main flaws is that they are unstable in the anodic potential window and exhibit an irreversible oxidation peak at roughly 0.43 V. Thus, combining MXenes with other components is essential for further enhancement of sensory abilities [204].

### 7.1. Nitrite Sensors

Nitrite (NO_2_^−^) is one of the most prevalent nitrogen-containing ions, which is widely used in the chemical and food industries [205,206]. Detection of nitrite is important as it poses serious dangers for human health. The nitrite oxidizes hemoglobin into methemoglobin, reducing the blood’s ability to carry oxygen [207], and it can also react to form N-nitrosamine, which can cause stomach cancer [208]. Tan Wang et al. created a novel nanocomposite of AuNPs/Ti_3_C_2_T_x_/ERGO for the sensitive electrochemical detection of nitrite. AuNPs/MXene/ERGO exhibited enhanced catalysis for nitrite oxidation compared to ERGO, MXENE/ERGO, and AuNPs/ERGO due to the synergy of these components. The developed sensor’s overall electrocatalytic oxidation of nitrite followed the mechanism:

NO_2_^−^ + H_2_O → NO_3_^−^ + 2H^+^ + 2e^−^

**Figure 9:**
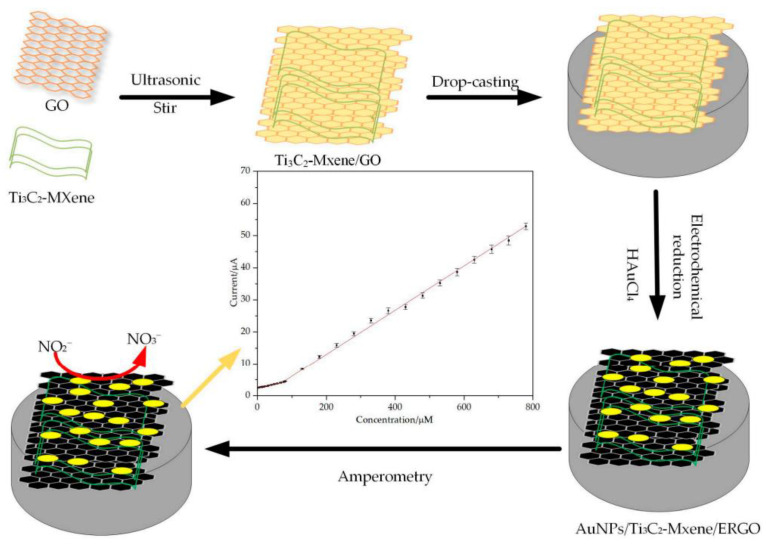
Nitrite detection scheme of AuNPs/MXene/ERGO-based sensor. Reprinted from [209].

Figure 9 shows the detection scheme using the obtained sensor. Linear ranges of obtained sensors were 0.5 to 80 µM and 80 to 780 µM, respectively, with an LOD of 0.15 µM and 0.015 µM and a sensitivity of 340.14 and 977.89 µA·mM^−1^cm^−2^ with great selectivity to nitrite (the sensor did not react to different interference salts, like KCl and NaNO_3_, even when their concentrations were much higher) [209].

The synergetic catalytic effect of the AuNPs/Ti_3_C_2_T_x_-PDDA nitrite sensor was designed by Yuhuan Wang et al. The Ti_3_C_2_T_x_ improved electron transport, and Ti_3_C_2_T_x_-PDDA composites gave AuNPs more places to attach during the electrodeposition process. The AuNPs/Ti_3_C_2_T_x_-PDDA/GCE sensor demonstrated exceptional linear ranges in the ranges of 0.1–2490 µM and 2490–13490 µM, high sensitivity of 250 µA·mM^−1^·cm^−2^, and great selectivity with an LOD of 0.059 µM. The sensor had a superior working range length and detection limit compared to a combination of AuNPs with graphene or CNT [210]. In order to combine the great electrocatalytic activity of AuNPs with carbon quantum dots (CQDs), MXene’s substantial specific surface area, and the good electrical conductivity of AuNPs with MXene, Xiwen Feng et al. created a AuNPs@CQDs-Ti_3_C_2_T_x_ MXene nitrite sensor, where MXene was used as the immobilization matrix. The sensor had a detection limit of 0.078 µM and a linear detection range of 1 µM to 3200 µM with great sensitivity and selectivity, as the sensor did not respond towards nitrates, chlorides and sulphates. However, it is worth noting that working range and detection limit were inferior to the AuNPs/Ti_3_C_2_T_x_-PDDA sensor [211]. A novel electrochemical sensing platform for nitrite detection was developed by Jinghao Zhuang et al. It was based on a Ti_3_C_2_T_x_ MXene sheet and a three-dimensional urchin-like Co(VO_3_)_2_(H_2_O)_4_ (CoVO). The following was proposed as the mechanism for the electrochemical reduction of nitrite on CoVO/Ti_3_C_2_T_x_ electrodes:

Co^2+^ ↔ Co^3+^ + e^−^

2Co^3+^ + NO_2_^−^ + H_2_O → 2Co^2+^ + NO_3_^−^ + 2H^+^

The CoVO/Ti_3_C_2_T_x_-based composite displayed a significantly improved electrochemical signal with good linearity in the range of 0.5–50 µM and 50–2000 µM with an LOD of 0.1 µM [212].

### 7.2. Hydrazine Sensors

Hydrazine and its derivatives are widely used in fuel cells, photography, insecticides, dyes, and pharmaceuticals [213]. Hydrazine is a known carcinogen and exposure to it can cause temporary blindness, skin allergies, carcinogenesis, neurotoxicity, and organ damage, especially to the liver and kidneys [214]. Additionally, industrial waste and waterways contain it, making it a potentially widespread environmental pollutant [213]. Therefore, creating efficient methods to detect hydrazine at low concentrations is essential from an industrial, medical, and environmental perspective. The idea of using few-layered (FL) Cr_2_CT_x_ MXene nanosheets as a receptor integrated into a microresonator to detect hydrazine at the ppm level was proposed by Bhuvaneswari Soundiraraju et al. [215]. The same author designed a FL-Cr_2_CT_x_ modified electrode for hydrazine detection. With an LOD of 6.60 × 10^−7^ M (at S/N = 3), the electrode demonstrated a high sensitivity of 1.810 µA·µM^−1^ towards hydrazine. The LOD for hydrazine was also more than 10 times lower than that achieved by electrodes modified by Ti_3_C_2_T_x_ and Nb_2_CT_x_-based pristine MXenes. The authors also presented a possible mechanism for detecting hydrazine by the sensor:

2 Cr_2_CO_x_ (surface) + N_2_H_4_ → 2Cr_2_CO_x−1_ + N_2_ + 2 H_2_O

The developed sensor was also employed to quantitatively analyze hydrazine liquid propellant spiked in water with satisfying results [216]. Yanqing Yao et al. intercalated a Ti_3_C_2_T_x_-based MXene as a conductive platform, which dramatically increased the conductivity of ZIF-8. The obtained sensor demonstrated excellent sensing performance for hydrazine, with amazing selectivity (with no reaction to nitrates, chlorides, ethanol, DMF and other possible interferences), a 10–7700 µM linear range, and an LOD of 5.1 µM, thanks to the synergistic effect of MXene/ZIF-8 nanocomposite. Compared to other sensors, the MXene/ZIF-8 sensor had a superior linear range, but it lacked a sufficient LOD [217]. Sundus Gul et al. used Nb_2_C MXene as a direct electrode material for electrochemical hydrazine sensing. They also added erbium (Er) to several Nb_2_C MXene samples and compared the results with non-doped ones. The pure MXene and Er-doped samples were found to have sensitivity values of 169 µAm^−1^·M^−1^·cm^−2^ and 276 µAm^−1^·M^−1^·cm^−2^ and LOD values of 10.08 mM and 67 µM, respectively, demonstrating that the Er-doped MXene is significantly more sensitive than the unmodified one. According to the authors both mentioned MXenes are potential candidates for hydrazine sensing [218].

### 7.3. Sensing of Phenol and Its Derivatives

The US Environmental Protection Agency and the European Union have identified phenolic compounds as environmental contaminants [219]. Phenolic compounds are common waste products of many chemical facilities. Bisphenols are frequently employed in a range of industries, along with benzenediols and aminophenols. Many of these compounds, even in very low quantities, are harmful to people, plants, animals, and biological and ecological ecosystems [220]. Thus, developing sensors to monitor the aforementioned substances is important to ensure the health of society and the environment. Lingxia Wu et al. described the application of Ti_3_C_2_ in phenol sensing [221]. According to Ling Lei et al., Ti_3_C_2_T_x_ MXene nanosheets are significantly more active for the oxidation of 4-chlorophenol (4-CP) and 4-nitrophenol (4-NP) than electrodes made from graphite. The linear ranges for 4-CP and 4-NP detection were 0.1 to 20.0 µM and 0.5 to 25.0 µM with detection limits of 0.062 µM and 0.11 µM, respectively. Though the sensitivity and LOD were significantly better than that of other electrodes, the linear range and stability were still lower in comparison to them. The sensor created by the authors was successfully applied to detect 4-CP and 4-NP in wastewater samples with promising results [222]. The exfoliated Ti_3_AlC_2_ was applied in sensor design, without oxidation, and the results demonstrated improved sensitivity of 16.35 µA·µM^−1^·cm^−2^ and a lower detection limit of 42 nM/L for 4-NP. With an excellent linear sensing range, MXene sensor electrodes were capable of detecting 4-NP in a broad concentration range from 500 nM to 100 M. The selectivity was also high, as the electrode did not react to phenol and its derivatives, like chlorophenol. The characteristics of the sensor were far superior in comparison to graphene-based sensors. The possible mechanism behind the 4-NP oxidation by Ti_3_C_2_T_x_ was also described as: 
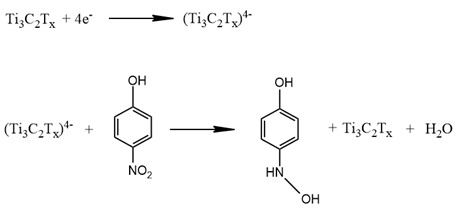


The sensors have also successfully shown reliability and repeatability in continuous monitoring of 4-NP in tap water samples with low interferences [223]. A new heterostructure made of Nb_2_CT_x_ and a Zn-Co-NC-based nanocage was proposed by Runmin Huang et al. The performance of the Nb_2_CT_x_/Zn-Co-NC-based sensor in the detection 4-NP was advanced by the large specific surface area and conductivity of MXene and the enhanced catalytic activity of the Nb_2_CT_x_/Zn-Co-NC-based nanocages. The linear range of the sensor was 1 µM to 500 µM, the LOD was 0.070 µM, and the sensitivity was 4.65 µA·µM^−1^·cm^−2^. The repeatability of the sensor was also studied, and it was shown that the relative standard deviation (RSD) was less than 3.48% [224].

The P. Abdul Rasheed group used a platinum nanoparticle/Ti_3_C_2_T_x_ nanocomposite to measure another phenol derivative—Bisphenol A—and to improve the electrochemical performance and stability of MXene used for the modification of electrodes. The sensor’s mechanism involves the oxidation of Bisphenol A [225]: 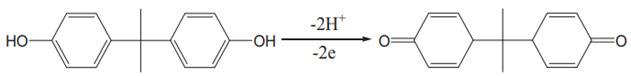


The proposed sensor had an extremely low LOD (32 nM) in a linear range of 0.05–5 µM, and it was further demonstrated to be successful in detecting Bisphenol A in samples of fresh milk and drinking water [225]. For efficient catechol and hydroquinone detection, Runmin Huang et al. decorated alkalization-intercalated Ti_3_C_2_ with N-doped porous carbon produced from MOFs. This strategy successfully stopped the Ti_3_C_2_ sheets from stacking and allowed benzenediol detection, which was based on hydrogen-bond interaction, due to the abundance of C-N and -OH functional groups. The sensor was characterized by a broad linear range of 0.5–150 µM with low detection limits of 4.8 nM (S/N = 3) for hydroquinone and 3.1 nM (S/N = 3) for catechol. The sensing mechanism revolved around oxidation of hydroquinone and catechol into quinone and 1,2-benzoquinone, respectively. The selectivity of the obtained sensor was high, with an RSD less than 6.6 from a large list of possible interferences (from ions to other phenols like bisphenol A and resorcinol). Compared to sensors from other materials, the obtained one had an adequate linear range and one of the lowest detection limits. Catechol and hydroquinone were also detected by the authors using the acquired sensor in industrial effluent with good recoveries [226].

### 7.4. Pesticide Sensors

Although pesticide residues pose a serious risk to human health, pesticides play a significant role in agricultural fields; hence, it is necessary to develop sensitive and quick methods for pesticide monitoring. For the purpose of determining the pesticide carbendazim (CBZ), Xiaolong Tu et al. developed a nanoarchitecture of Ti_3_C_2_T_x_/CNHs (carbon nanohorns)/-CD-MOFs (cyclodextrin–metal–organic frameworks). Due to the synergistic combination of the high electronic conductivity and better catalytic activity from MXene/CNHs and the plentiful electrocatalytic active sites from -CD-MOFs, the MXene/CNHs/-CD-MOFs demonstrated boosted catalytic activity toward CBZ oxidation. The mechanism behind CBZ sensing involved oxidation of the compound on the sensor: 
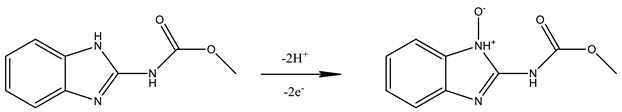


A large linear range from 3.0 nM to 10.0 µM and a low LOD of 1.0 nM (S/N = 3) were both determined for the MXene/CNHs/-CD-MOF electrode. The produced sensor was then used in tomato samples, exhibiting high selectivity, repeatability, and long-term stability [227]. Yu Xie et al. used MXene/ERGO composite for CBZ detection to enhance the Ti_3_C_2_T_x_ sensing capability. The separated Ti_3_C_2_T_x_ layers and particles were tightly connected by the ERGO conductive networks, thus improving the electrochemical reactivity of the electrode. The electrochemical sensor was characterized by a low LOD of 0.67 nM and a large linear range of 2.0 nM to 10.0 µM and was used to measure CBZ in food products [228]. Prussian blue (PB) and Ti_3_C_2_T_x_ hybrid composites for H_2_O_2_ and malathion sensing were created by Ying He et al. In the sensor, Ti_3_C_2_T_x_ served as a reducing agent, and the multilayer 2D nanostructure efficiently prevented PB nanoparticles from aggregating. With a detection limit of 1.3 × 10^−16^ M, good linearity of malathion was obtained in the range of 1.0 × 10^−15^ to 1.0 × 10^−9^ M [229]. Fengnian Zhao et al. created Au-Pd bimetallic nanoparticles through a self-reduction process using ultrathin MXene nanosheets as the reducing agent and support. These nanoparticles advanced the immobilization of acetylcholinesterase and the determination of paraoxon. The MXene/Au-Pd biosensor with an LOD of 1.75 ng·L^−1^ showed a good linear relationship with paraoxon concentration from 0.1 to 1000 µg·L^−1^, and promising results with cucumber samples were obtained [230]. A new acetylcholinesterase biosensor based on Ti_3_C_2_T_x_ nanosheets and chitosan was developed by Liya Zhou et al., and it was found to be suitable for the detection of organophosphate pesticides. The biosensor performed well in terms of malathion detection, with a linearity in the range of 1 × 10^−14^–1 × 10^−8^ M and an LOD of 0.3 × 10^−14^ M [231].

## 8. Optical Sensing

Optical sensing is a promising approach for the detection of chemical compounds because of its high accuracy, strong anti-interference ability, and the non-invasive nature of the measurement [105]. Ti_3_C_2_T_x_-based MXene has a variety of intriguing characteristics that can benefit from optical sensing [232,233,234]. Ti_3_C_2_T_x_ has strong wavelength dependence in its optical absorption zone (from the visible to the short-wave infrared) [235]. In addition, Ti_3_C_2_T_x_ has a high intrinsic photothermal conversion efficiency in the infrared irradiation range, allowing the absorbed irradiation power to produce heat effectively [236]. The photoelectric properties of MXenes can also be easily controlled by surface modification [237]. Wide-band optical absorption and good optical modulation are the result of the direct band gap structure of MXene, which may be altered by changing surface functional groups or the number of layers [238,239].

Electrochemiluminescence (ECL) is a sensitive analytical technique that combines the benefits of electrochemistry and luminescence [240,241,242]. Electrochemically produced intermediates in ECL go through a highly exergonic process to create an excited state, which eventually relaxes to a lower-level state with the emission of light. ECL offers great sensitivity, low cost, and a wide dynamic range; therefore, it can be integrated within simple, easily controllable, and portable analytical instrumentation [243,244]. A prominent biomarker for the diagnosis of chronic myelogenous leukemia is the oncogenic BCR-ABL fusion gene, a distinctive gene of the Ph chromosome [245]. Heterojunction based on CeO_2_/Ti_3_C_2_T_x_ was applied to an ECL biosensor for the detection of BCR-ABL. The synthesized heterojunction with strong dispersibility and a large specific surface area synergistically increased the ECL signal of the S_2_O_8_^2−^/O_2_ system and improved the immobilization of biomolecules. The sensor demonstrated a large working concentration range from 1 fM to 100 pM [246].

The process of photoelectrochemistry, or PEC, is defined as the interaction of optical and electrochemical processes wherein an applied light causes electron excitation after charge transfer via photoexcited material [247,248]. The PEC technique uses light as the detecting signal and photocurrent excitation, which is the opposite to ECL [249]. The advantages of sensing based on PEC are the low background signal, easy setup, and excellent sensitivity [250,251]. The PEC-SERS dual-mode biosensor was developed by Fanglei Liu et al. to detect *Staphylococcus aureus* with high accuracy and sensitivity. The method made use of the superior PEC and SERS modes of carbon nitride nanosheet (C_3_N_4_)/Ti_3_C_2_T_x_-AuNPs. According to experimental findings, PEC and SERS work well together to amplify *Staphylococcus aureus* detection, with detection ranges of 5–10^8^ CFU/mL and an LOD of 0.70 CFU/mL for PEC, and 10–10^8^ CFU/mL and 1.35 CFU/mL for SERS, respectively [252].

The measurement of fluorescence intensity is mostly applied during the action of fluorescence sensors. Typically, these sensors yield low LOD and high sensitivity. Fungal toxins are very dangerous substances due to their mutagenicity, teratogenicity, carcinogenicity, and other characteristics [253,254]. Therefore, the development of efficient methods for toxin detection is crucial, as the potential contamination of cereals, peanuts, and maize puts human and animal health at risk [255]. A unique fluorescent biosensor for Aflatoxin B1 (AFB1) detection based on the CRISPR/Cas12a system deposited on Ti_3_C_2_T_x_ was created by Zhihui Wu et al. When AFB1 was present, the aptamer’s preferential binding to it released the activator, which in turn triggered Cas12a’s trans-cleavage activity, which indiscriminately cleaved ssDNA on MXenes and restored the fluorescence signal. With a detection range of 0.001 to 80 ng·mL^−1^ and a detection limit of 0.92 pg·mL^−1^, the fluorescent biosensor was highly versatile [256].

Optical-calorimetry-based techniques are mostly either quantitative or semiquantitative due to their moderate LODs [257], with principal advantages being their inexpensive construction costs and ease of use. Yapeng Li et al. manufactured Ti_3_C_2_/CuS nanocomposites and used them in a sensor for the detection of cholesterol. When utilized as a peroxidase to catalyze the reaction of 3,3,5,5-tetramethylbenzidine (TMB) in the presence of H_2_O_2_, MXene-based nanocomposites demonstrated peroxidase-like activity and caused a change in color to blue. The cholesterol determination using nanocomposites exhibited a linear range between 10 and 100 μM and LOD of 1.9 μM. Results of the same research illustrated that nanocomposites based on MXene-Ti_3_C_2_/CuS can be applied in clinical medicine for the detection of H_2_O_2_ and cholesterol [258].

Surface plasmon resonance (SPR) is a process in which photons of incident light at a specific angle excite the electrons in the metallic surface layer, which then travel parallel to the metal surface [259,260,261]. The refractive index, or RI, of the substance close to the metal surface determines the specific angle that initiates SPR when there is a constant light source frequency and a thin metal surface. Various analytes can be detected by this technique since a slight alteration in the sensing medium’s reflective index will prevent SPR from occurring from a certain angle [262]. Thiol-modified Nb_2_C quantum dots (Nb_2_C-SH QDs) have been developed by Rongyuan Chen et al. for use in a novel label-free SPR aptasensor for the detection of SARS-CoV2 N-gene. Nb_2_C-SH QDs improved the SPR response in addition to having a high bioaffinity for the aptamer. The SPR aptasensor demonstrated a low LOD of 4.9 pg·mL^−1^ sensitivity in the concentration range of 0.05 to 100 ng·mL^−1^. Additionally, a qualitative examination of N-gene from various sources, such as human serum, seafood, and seawater, was demonstrated using the aptasensor [263].

Raman scattering spectroscopy has distinct advantages over other spectroscopy techniques, including narrow peaks in multicomponent analysis and frequency shifts that offer “fingerprint” information about the chemical structure of an analyte [264]. Nevertheless, the limited sensitivity of Raman spectroscopy renders it useless for the identification of clinical indicators. Surface-enhanced Raman scattering (SERS) occurs when molecules are adsorbed on or near rough metal surfaces or metal-based nanostructures and exhibit an amplification in their Raman signal [262]. To combine the benefits of MXene and metal nanoparticles for SERS, Rongyang Liu et al. created a hybrid biosensor, Ti_3_C_2_T_x_-AgNPs. Dopamine and adenine monitoring demonstrated the SERS sensor’s capacity to identify biological molecules; the LOD for adenine was 10^−8^ M, while the LOD for dopamine was 5 × 10^−8^ M. The biosensor’s capacity for detection in serum samples was further demonstrated [265].

## 9. Conclusions and Outlook

MXenes offer a myriad of advantages that have led to the development of various sensors, including strain, pressure, gas, electrochemical, optical, humidity, and multifunctional sensors. However, there are challenges and issues that need to be addressed for the optimal utilization of MXenes in sensor design. Some of the notable issues include specificity and selectivity, integration within other materials, stability and durability, cost-effectiveness, advanced manufacturing techniques, and difficulties in standardization. One of the challenging issues that needs to be resolved before effectively utilizing MXenes in sensor design is enhancing the specificity and selectivity of MXene-based sensors for certain target analytes. Additionally, achieving high sensitivity without compromising on the ability to discriminate between different substances is crucial for the reliability of the sensors. While MXenes exhibit excellent properties, combining them with other materials enables the achievement of the desired sensor characteristics. However, ensuring compatibility and efficient integration with other components is still a key consideration for practical sensor designs. The long-term stability and durability of MXene-based sensors, especially under harsh environmental conditions or repeated usage, need further improvement. Ensuring that the sensors maintain their performance over extended periods is essential for practical applications. The production cost of MXenes can be a limiting factor in large-scale applications. Research efforts are ongoing to develop cost-effective methods for MXene synthesis and processing to make them more accessible for widespread sensor deployment; however, at the current time, MXenes remain very expensive materials. Current manufacturing methods for MXene-based sensors involve complex processes. Therefore, streamlining and optimizing these techniques are essential for mass production and commercialization of MXene-based sensors. Further establishing standardized protocols for MXene-based sensor fabrication and testing is crucial for ensuring consistency and comparability across different research studies and applications. The environmental impact of MXene production and disposal is still under consideration. Therefore, a high number of sustainable and eco-friendly approaches for MXene synthesis and integration into sensors are being explored. It is still currently difficult to synthesize MXene-based sensing materials in a controlled manner. For instance, producing MXene nanosheets with specific parameters or surface terminations (currently, a lot of methods result in an abundance of -F groups, which can be damaging to human health and the environment) is still challenging. To this day, the most popular pathways to synthesize MXenes require using or producing hazardous chemicals. Secondly, the chemical stability of MXenes (especially in aqueous solutions) also needs further research and improvements, as the oxidation of MXenes (especially, Ti_3_C_2_T_x_) hinders the progress in many applications (for example, biosensors). Finally, more different materials that belong to the MXene family could be applied in sensor design as V-, Mo-, Zr-, Nb-based MXenes and many others can provide a lot of benefits for certain types of sensors, compared to the most commonly used Ti_3_C_2_T_x_-based MXene. Addressing these challenges will contribute to the continued advancement and widespread adoption of MXene-based sensors in various industries. Ongoing research and collaborative efforts are key to overcoming these issues and unlocking the full potential of MXenes in sensor design. Therefore, it is expected that a great number of MXene-based sensors, such as strain, pressure, gas, electrochemical, optical, humidity, and even multifunctional sensors, will be developed by taking advantage of their features, which includes their great electrical conductivity, tunable surface functional groups, a great surface area, superior electrochemical and electrocatalytic capabilities, and remarkable optical properties.

## Figures and Tables

**Table 1:** MXene application in strain sensors.

Composition	Sensitivity (GF)	Test Range, Strain %	LOD, Strain %	Ref
Ti_3_C_2_T_x_/MWCNTs/TPU	13–363	0–100	-	[58]
Ti_3_C_2_T_x_ MXene/polyurethane	22.9–228	58–150	0.1	[59]
CNT/Ti_3_C_2_T_x_ MXene/PDMS	13.3	0–60.3	-	[53]
MXene/PANIF	97.6–2369.1	0–80	0.1538	[60]
Ti_3_C_2_T_x_ MXene/paper	17.4	0–0.6	0.1	[61]
Ti_3_C_2_T_x_ MXene/P(VDF-TrFE)	6.35–108.8	45–66	-	[62]
PVA/CMC/TA/MXene hydrogel	2.9	0–700	1	[63]
PVA-CA-MXene hydrogel	2.3	0–200	-	[64]
Polyvinyl alcohol/polyacrylamide/CaCl_2_/MXene	3.0	0–300	-	[65]
Liquid metal/MXene	7.85–15.47	0–400	-	[66]
PAA/PEDOT:PSS/MXene hydrogel	9–20.86	0–1000	-	[67]
MXene/AgNWs/TPU	33.1	0–120	-	[68]

**Table 2:** MXene application in pressure sensors.

Composition	Sensitivity, kPa^−1^	Test Range, kPa	LOD, Pa	Ref
MXene/CTAB/CMF	381.91	0−3	0.1	[95]
MXene/PPNs/MXene/TPUEM	34.9–177.3	0–30.4	-	[96]
MXene/Polyetherimide	20 (150 °C)–80 (−5 °C)	0–40	9	[97]
MXene/rGO/PS	115–224	0–20.65	-	[98]
MXene/bacterial cellulose (BC)	0.94–95.2	0–10	0.4	[99]

## Data Availability

No new data were created or analyzed in this study. Data sharing is not applicable to this article.

## Abbreviations

VOC	volatile organic compound
LOD	limit of detection
CNT	carbon nanotube
NW	nanowire
PPy	polypyrrole
HEC	hydroxyethyl cellulose
MWCNTs	multi-walled carbon nanotubes
TPU	thermoplastic polyurethane
PDMS	polydimethylsiloxane
PANI	polyaniline
PANIF	polyaniline fiber
P(VDF-TrFE)	poly(vinylidenefluoride-co-trifluoroethylene)
PVA	polyvinyl alcohol
CMC	sodium carboxymethylcellulose
TA	tannic acid
PVA-CA	catechol-functionalized poly(vinyl alcohol)
PAA	polyacrylic acid
PEDOT	poly(3,4-ethylenedioxythiophene)
PSS	poly(styrene-sulfonate)
ANFs	aramid nanofibers
ZIF-67	zeolitic imidazolate framework-67
PAN	polyacrylonitrile
PE	polyethylene
CTAB	hexadecyl trimethyl ammonium bromide
CMF	carbon sponge
PPNs	polypyrrole nanospheres
TPUEM	thermoplastic polyurethane electrospinning membrane
GO	graphene oxide
rGO	reduced graphene oxide
PS	polystyrene
BC	bacterial cellulose
CA	κ-carrageenan
PAM	polyacrylamide
PHOS	photothermal optical sensor
MZI	Mach–Zehnder interferometer
MKR	micro-fiber knot resonator
RH	relative humidity
TOCNFs	(TEMPO)-oxidized cellulose nanofibers
SAW	surface acoustic wave
MEMS	micro-electromechanical systems
NPs	nanoparticles
ERGO	electrochemically reduced graphene oxide
PDDA	poly(dimethyl diallyl ammonium chloride)
GCE	glassy carbon electrode
CQDs	carbon quantum dots
FL-Cr_2_CT_x_	few-layered two-dimensional chromium carbide
ZIF-8	zeolitic imidazolate framework-8
CC	carbon cloth
4-CP	4-chlorophenol
4-NP	4-nitrophenol
NC	nanocage
RSD	relative standard deviation
MOF	metal–organic framework
CBZ	carbendazim
CNHs	carbon nanohorns
CD-MOFs	cyclodextrin–metal–organic frameworks
PB	Prussian blue
ECL	electrochemiluminescence
PEC	photoelectrochemistry
CFU	Colony-forming unit
CRISPR	clustered regularly interspaced short palindromic repeats
TMB	3,3,5,5-tetramethylbenzidine
SPR	surface plasmon resonance
SERS	surface-enhanced Raman scattering
